# Guidelines for genetic testing in prostate cancer: a scoping review

**DOI:** 10.1038/s41391-023-00676-0

**Published:** 2023-05-18

**Authors:** Haitham Tuffaha, Kim Edmunds, David Fairbairn, Matthew J. Roberts, Suzanne Chambers, David P. Smith, Lisa Horvath, Shiksha Arora, Paul Scuffham

**Affiliations:** 1 Centre for the Business and Economics of Health, University of Queensland, Brisbane, QLD Australia; 2 Pathology Queensland, The Royal Brisbane Women’s Hospital, Brisbane, QLD Australia; 3 UQ Centre for Clinical Research, University of Queensland, Brisbane, QLD Australia; 4 Department of Urology, Royal Brisbane and Women’s Hospital, Brisbane, QLD Australia; 5 The Faculty of Health Sciences, Australian Catholic University, Brisbane, NSW Australia; 6 The Daffodil Centre, The University of Sydney, A Joint Venture with Cancer Council NSW, Sydney, NSW Australia; 7 Medical Oncology, Chris O’Brien Lifehouse, Camperdown, NSW Australia; 8 Clinical Prostate Cancer Group, Garvan Institute of Medical Research, Darlinghurst, NSW Australia; 9 Faculty of Medicine and Health, University of Sydney, Camperdown, NSW Australia; 10 Menzies Health Institute Queensland, Griffith University, Gold Coast, QLD Australia

**Keywords:** Cancer genetics, Cancer genetics

## Abstract

**Background:**

Genetic testing, to identify pathogenic or likely pathogenic variants in prostate cancer, is valuable in guiding treatment decisions for men with prostate cancer and to inform cancer prevention and early detection options for their immediate blood relatives. There are various guidelines and consensus statements for genetic testing in prostate cancer. Our aim is to review genetic testing recommendations across current guidelines and consensus statements and the level of evidence supporting those recommendations.

**Methods:**

A scoping review was conducted following the Preferred Reporting Items for Systematic Reviews and Meta-analyses extension for scoping review (PRISMA-ScR) guidelines. Electronic database searches and manual searches of grey literature, including websites of key organisations were conducted. Using the Population, Concept, Context (PCC) framework, this scoping review included: men with prostate cancer or men at high risk of prostate cancer and their biological families; existing guidelines and consensus statements with supporting evidence for genetic testing of men with prostate cancer from any geographical location worldwide.

**Results:**

Of the 660 citations identified, 23 guidelines and consensus statements met the inclusion criteria for the scoping review. Based on different levels of evidence about who should be tested and how, a diverse range of recommendations were identified. There was general consensus among the guidelines and consensus statements that men with metastatic disease be offered genetic testing; however, there was less consensus in relation to genetic testing in localised prostate cancer. While there was some consensus in relation to which genes to test, recommendations varied regarding who to test, testing methods and implementation.

**Conclusion:**

While genetic testing in prostate cancer is routinely recommended and numerous guidelines exist, there is still considerable lack of consensus regarding who should be tested and how they should be tested. Further evidence is needed to inform value-based genetic testing strategies for implementation in practice.

## Introduction

Prostate cancer is the third most diagnosed cancer worldwide and the second most commonly diagnosed amongst men after lung cancer. Around 1.4 million new cases and 0.4 million deaths were reported in 2020 due to prostate cancer [1]. While earlier detection due to prostate-specific antigen (PSA) screening contributed to improved survival outcomes, it also increased the economic burden of prostate cancer through overdiagnosis and further testing [2]. Prostate cancer is a multifactorial and heterogenous cancer and while the cost of prostate cancer treatment varies across countries [2], costs are increasing more rapidly than those of any other cancer [3]. The incidence of metastatic cancer is also increasing in populations worldwide, particularly in younger populations, with the potential to contribute to a 40% increase in the annual burden by 2025 [3]. In Australia, for example, prostate cancer is the most common cancer in men (>24,000 cases diagnosed in 2022) and a leading cause of cancer-related deaths (~3500 a year or ~22 deaths per 100,000 males) [4]. The estimated annual cost of prostate cancer treatment to Australia (2015–2016) is approximately $684 million [5], and projected to increase considerably over the next 10 years [6]. Personalised prevention and treatment has the potential to improve the efficiency of healthcare and mitigate some of these costs [7].

Prostate cancer has a strong genetic component [8–12]. The proportion of prostate cancer attributable to hereditary factors is estimated to be between 5 and 15% [13]. For example, up to 15% of men with metastatic and 10% in men with localised prostate cancer have mutations in homologous recombination repair (HRR) genes, such as *BRCA2, BRCA1, ATM, CHEK2, PALB2*, and mismatch repair (MMR) genes (*MLH1, MSH2, PMS2* and *MSH6*). Several inherited mutations (e.g., *BRCA1* and *BRCA2*) are associated with varying degrees of increased predisposition to prostate cancer [8–12]. These mutations are linked with a younger age of cancer onset, an aggressive clinical course, and increased cancer mortality [14]. Genetic testing, including germline testing of hereditary cancer risk, can inform treatment decisions for men with prostate cancer as well as cancer risk in healthy individuals [15, 16]. Targeted therapies such as poly (ADP-ribose) polymerase (PARP) inhibitors (e.g., olaparib and rucaparib) are approved in multiple jurisdictions for the treatment of men with metastatic castration-resistant prostate cancer (mCRPC) who carry mutations in *BRCA1* and *BRCA2*, based on the pivotal PROfound and TRITON2 clinical trials [17, 18]. Furthermore, men who are identified to carry *BRCA* mutations could benefit from prostate cancer screening at an early age (e.g., forty years) [19]. Importantly, germline testing can reveal higher risk of hereditary cancers including hereditary breast and ovarian cancer (HBOC) syndrome with *BRCA1* and *BRCA2* mutations, and Lynch syndrome with mutations in MMR genes including *MLH1, MSH2, PMS2* and *MSH6* [20].

With the increasing importance of genetic testing in prostate cancer, a number of clinical practice guidelines and consensus statements have been developed by multiple professional organisations (e.g., National Comprehensive Cancer Network (NCCN) [21]; European Association of Urology (EAU) [22]; European Society for Medical Oncology (ESMO) [23]; and Philadelphia Prostate Cancer Consensus Conference [16]. Given the large number of men who could potentially be eligible for testing, these guidelines and consensus statements provide risk-based genetic testing criteria which encompass personal and disease factors (e.g., cancer history and disease stage) together with family history and ancestry (e.g., Ashkenazi Jewish ancestry). Nevertheless, since genetic testing in prostate cancer is a rapidly evolving field and the evidence base to inform genetic testing recommendations (i.e., who should be tested and how) is underdeveloped, there are differences in the genetic testing recommendations across the guidelines and consensus statements. Reviewing current genetic testing criteria and how these vary across guidelines and consensus statements is important to highlight areas of discrepancy and identify the gaps in existing evidence to guide future research efforts. To date, there is no published comprehensive review of genetic testing recommendations in prostate cancer. Therefore, the objectives of this scoping review are to identify and compare: 1) current genetic testing recommendations in terms of who should be tested, for which genes and how they should be tested, and 2) the level of evidence used in supporting those recommendations.

## Materials and methods

A scoping review protocol was developed based on the Arksey and O’Malley [24] and Peters et al. [25] methodological frameworks and the Preferred Reporting Items for Systematic Reviews and Meta-analyses extension for scoping review (PRISMA-ScR) Statement [26]. The protocol included a systematic process for conducting the literature search including study/guideline selection, data charting, summarising and reporting results. The protocol can be accessed in Appendix I.

### Search strategy

A preliminary search of MEDLINE, the Cochrane Database of Systematic Reviews and JBI Evidence Synthesis revealed no systematic or scoping reviews on genetic testing for prostate cancer guidelines and consensus statements. Therefore, an initial limited search of MEDLINE and CINAHL was undertaken to identify relevant articles to inform the search strategy. The index terms and text words contained in the titles and abstracts of relevant articles were used to develop a full search strategy for genetic testing guidelines and consensus statements for prostate cancer in consultation with the research team and senior health sciences librarian. The aim of the search strategy, outlined in Appendix II, was to locate both published and unpublished guidelines and consensus statements. We searched four electronic databases (PubMed, Embase, CINAHL, PsycInfo) and the grey literature, including websites of key organisations (e.g., NCCN, EAU, AUA (American Urology Association), ESMO, eviQ). Guidelines and consensus statements published since April 1, 2007, when the first genome wide association study for prostate cancer was published, until May 30 2022, were included to ensure all possible guidelines and consensus statements and associated evidence were captured [27]. The reference list of all included sources of evidence was screened and, given the burgeoning interest in genetic testing, database alerts (May 31, 2022 - August 5, 2022) were set up to capture new guidelines or consensus statements for genetic testing in prostate cancer after the original search was completed.

Using the Population, Concept, Context (PCC) framework (Table 1), strict eligibility criteria were followed when selecting sources of information:

**Table 1:** Eligibility criteria: population, concept, context.

Eligibility criteria	Inclusion criteria	Exclusion criteria
**P**	Prostate cancer patients or men at high-risk of prostate cancer and their families	<18 yrs; men without prostate cancer and no/low risk
**C**	Recommendations, guidelines, consensus statements and supporting evidence for genetic testing of prostate cancer	Superseded guidelines or consensus statements, published papers such as opinion pieces, commentaries, editorials, conference abstracts. Guidelines or consensus statements not based on a rigorous methodology of consensus or adequate evidence
**C**	Any context where genetic testing for prostate cancer is possible. No specific cultural /sub-cultural factors, geographical locations, specific racial or gender-based considerations	

### Types of sources

#### Inclusions

Inclusion criteria were developed so all guidelines and consensus statements providing genetic testing recommendations for prostate cancer were considered. We defined a guideline or consensus statement as any evidence-based and consensus-based set of recommendations for genetic testing in prostate cancer involving stakeholders with relevant expertise or experience [28]. All major organisational guidelines and consensus statements were included whether published in journals or on websites (e.g., NCCN, ESMO, eviQ). These are regularly updated and provide a clear methodology around development and consensus processes and the expertise and evidence used to inform decisions. Strength of recommendation ratings were also included. Reviews of these major guidelines and consensus statements were included where they were conducted by a consortium or multidisciplinary national or international team and adapted with the aim of addressing gaps or developing country/region specific guidelines or consensus statements or to advance clinical application or implementation of guidelines or consensus statements. In order to capture all relevant guidelines and consensus statements, the context was intentionally broad.

#### Exclusions

Superseded guidelines and consensus statements or published papers such as opinion pieces, commentaries, editorials and conference abstracts were excluded.

#### Source of evidence selection

**Fig. 1:**
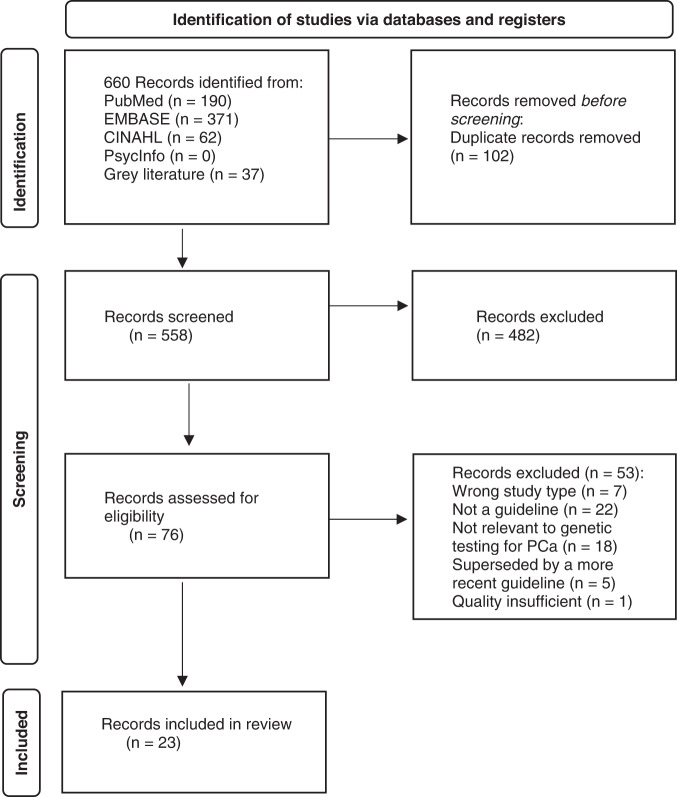
PRISMA diagram. The stages of the literature search process.

Following the search, all identified citations were collated and uploaded into Endnote 20 *(Clarivate Analytics, PA, USA)* and duplicates removed. The Endnote file was then uploaded into Covidence *(Veritas Health Innovation, Melbourne, Australia)*. Titles and abstracts were then screened by two independent reviewers for assessment against the inclusion criteria for the review. Potentially relevant sources were retrieved in full. The full text versions of selected citations were assessed in detail against the inclusion criteria by two independent reviewers. Reasons for exclusion were recorded for report in the scoping review. Any disagreements that arose between the reviewers at each stage of the selection process were resolved through discussion. The results of the search and the study inclusion process are presented in a PRISMA-ScR flow diagram (Fig. 1) [26].

#### Research questions

##### Six research questions informed the data extraction:

- What genetic testing guidelines and consensus statements for prostate cancer currently exist?
- What are the recommendations for genetic testing of prostate cancer?
- Who should be considered for genetic testing?
- Which genes should be tested for?
- Which testing methods are used and where are samples drawn from?
- What evidence supports the recommendations?

#### Data extraction

Data were extracted from papers included in the scoping review using a data extraction tool developed by the reviewers and included specific details about the guideline details: Organisation, year, country of origin, criteria for genetic testing for men at risk or at different stages of prostate cancer, recommended test and genes tested, and level and/or strength of evidence. To address heterogeneity in strength of recommendation ratings and facilitate comparison across guidelines, we mapped the rating instruments (excluding expert opinion only) used in different guidelines and consensus statements to the National Health and Medical Research Council (NHMRC) grades of recommendation (Table 2) [29].

**Table 2:** NHMRC grades of recommendation.

Grade	Strength	Explanation
A	Strong	Body of evidence can be trusted to guide practice
B	Strong/Moderate	Body of evidence can be trusted to guide practice in most situations
C	Moderate	Some support for recommendation/s but care should be taken in its application
D	Weak	Recommendation must be applied with caution

Summary data were then extracted for reporting in the scoping review (Table 3). Both reviewers extracted data from full text inclusions as quality assurance. Any disagreements were resolved through discussion.

**Table 3:** Summary table of major genetic testing for prostate cancer guidelines.

Guideline organisation Title (Year) Country	Criteria for genetic testing by cancer diagnosis					
	Men without PCa (Stage 0)^a^	Strength of Recom-mendation	Men with localised PCa (Stage I or II)^b^	Strength of Recom-mendation	Men with locally advanced or advanced PCa (Stage III or IV)^c^	Strength of Recom-mendation^d^
**Major guidelines**						
1. NCCN (2022) US a. Clinical Practice Guidelines in Oncology: Prostate v4.2022 b. Clinical Practice Guidelines in Oncology: Genetic/Familial High-Risk Assessment: Breast, Ovarian, and Pancreatic v2.2022 c. Clinical Practice Guidelines in Oncology: Genetic/Familial High-Risk Assessment: Colorectal v1.2022	**Germline testing** ▪ Known or suspected mutation in a cancer susceptibility gene within the family. ▪ Close blood relative meeting criteria for personal history of **breast cancer** and: ◦ ≤45y ◦ Multiple breast cancers ◦ ≥1 close blood relative with: - breast cancer ≤50y - male breast cancer - ovarian cancer - pancreatic cancer - metastatic, intraductal/ cribriform histology, high risk prostate cancer - >5% probability of *BRCA1/BRCA2* variant ▪ Close blood relative with epithelial **ovarian cancer** ▪ FDR diagnosed with exocrine **pancreatic cancer** ▪ FDR meeting criteria for personal history of **prostate cancer** and: ◦ metastatic, intraductal/ cribriform histology, high risk prostate cancer ◦ ≥1 close blood relative with: - breast cancer ≤50y - ovarian cancer - pancreatic cancer ◦ ≥2 close blood relatives with breast or prostate cancer ▪ Individual meeting any criteria above but tested negative with previous limited testing (e.g., single gene) interested in multi-gene testing ▪ Ashkenazi Jewish ancestry without additional risk factors ▪ Individual with LS should consider risk based on LS gene and family history of PCa and consider annual PCa screening at 40y	**Strong/ Moderate** for all recommen-dations	**Germline testing** ▪ Known or suspected mutation in a cancer susceptibility gene within the family (especially in *BRCA1, BRCA2 ATM, PALB2, CHEK2, MLH1, MSH2, MSH6, PMS2, EPCAM*) ▪ Personal history of **prostate cancer** and ◦ intermediate risk PCa and intraductal/cribriform histology ◦ a prior personal history of any of the following: - exocrine pancreatic, upper tract urothelial, glioblastoma, bilary tract & small intestinal ◦ ≥1 close relative with: - breast cancer ≤50y - colorectal or endometrial cancer ≤50y - male breast cancer - ovarian cancer - exocrine pancreatic cancer - metastatic, high risk PCa ◦ ≥1 FDR with PCa ≤60y ◦ ≥2 close relatives with breast or prostate cancer ◦ ≥3 close relatives with LS-related cancers (colorectal, endometrial, gastric, ovarian, exocrine pancreatic, upper tract urothelial, glioblastoma, biliary tract and small intestinal), especially if diagnosed ≤50y ◦ Ashkenazi Jewish ancestry ▪ Personal history of breast cancer **Somatic testing** ▪ Alterations in HRR genes (*BRCA1, BRCA2, ATM, PALB2, FANCA, RAD51D, CHEK2, and CDK12*) ▪ MSI-H or MMR genes ▪ FDR meeting any criteria listed above	**Strong/Moderate** for all recommen-dations	**Germline testing** ▪ High-risk, very-high-risk, regional (node positive) or metastatic PCa, regardless of family history or age ▪ Personal history of breast cancer ▪ Positive FH: ◦ ≥1 FDR, SDR, or TDR with breast, colorectal or endometrial cancer at age ≤50 years, male breast cancer, ovarian or exocrine pancreatic cancer, at any age; ◦ ≥1 FDR with PCa ≤60 years or who died from PCa; ◦ ≥2 close relatives with breast cancer or PCa at any age; ◦ ≥3 close relatives with LS-related cancers (colorectal, endometrial, gastric, ovarian, exocrine pancreas, upper tract, urothelial, glioblastoma, biliary tract, & small intestinal), especially if diagnosed at age <50 years ▪ Ashkenazi Jewish ancestry **Somatic testing** ▪ Men with mPCa ▪ Men with mCRPCa ▪ Homologous recombination gene mutations in men with regional N1 PCa ▪ MSI-H or MMR genes in men with regional or metastatic castration naive PCa ▪ TMB testing in men with mCRPCa.	**Strong/Moderate** for all recommen-dations
2. EAU – EANM – ESTRO – ESUR – ISUP – SIOG (2022) The Netherlands Guidelines on Prostate Cancer	**Germline testing** ▪ Men with multiple family members diagnosed with csPCa at age <60 years or family member PCa death; ▪ Men with family history (paternal and maternal) of high-risk germline mutations or multiple cancers on same side of family (e.g., *BRCA1/BRCA2, ATM, HOXB13*)	**Weak** **Weak**	**Germline testing** ▪ Men with multiple family members diagnosed with csPCa at age <60 years or family member PCa death; ▪ Men with FH of high-risk germline mutations or multiple cancers on same side of family.	**Weak** **Weak**	**Germline testing** ▪ Men with high-risk PCa and a family member diagnosed with csPCa at age <60 yrs **Somatic testing** ▪ Men with mPCa ▪ Men with mCRPCa	**Weak** **Weak** **Strong**
3. Philadelphia Prostate Cancer Consensus Conference US Implementation of Germline Testing for Prostate Cancer (**2020)** Role of genetic testing for inherited cancer risk **(2018)**	**Germline testing** ▪ ≥1 brother or father or ≥2 male relatives: ◦ diagnosed with PCa at <60 y; ◦ with mPCa; ◦ died from PCa. ▪ ≥2 or more cancers in HBOC or LS in any relatives, especially if diagnosed at age <50 y	NR	**Germline testing** ▪ Nonmetastatic PCa and one of the following: ◦ Locally advanced disease T3a or higher ◦ Intraductal/ductal pathology ◦ ≥ grade group 4 (Gleason 8) ◦ Ashkenazi Jewish ancestry ▪ ≥1 brother or father or ≥2 male relatives: ◦ diagnosed with PCa at <60 y; ◦ with mPCa; ◦ died from PCa. ▪ ≥2 or more cancers in HBOC or LS in any relatives, especially if diagnosed at age <50 y	NR	**Germline testing** ▪ mHSPCa or mCRPCa; ▪ Confirmatory germline testing for somatic mutations *BRCA2* ▪ Test additional genes on the basis of personal or family history **Somatic testing** ▪ Somatic next generation sequencing for all men with metastatic PCa	NR
4. AUA/ASTRO US Clinically Localised Prostate Cancer Part I Guideline **(2022)** AUA/ASTRO/ SUO US Advanced Prostate Cancer Guideline: Parts I & II **(2021)**	NA		**Germline testing** Localised Part 1 ▪ Strong family history of PCa: FDR or multiple SDRs diagnosed with grade 2 or higher PCa, particularly at early age (<60y), particularly if metastatic or lethal ▪ Strong personal or family history of related cancers: Breast, colorectal, ovarian, pancreatic, upper tract urothelial carcinoma ▪ Known family history of familial cancer risk mutation: e.g., BRCA1, BRACA2, ATM, LS associated genes ▪ Ashkenazi Jewish ancestry: Particularly those with grade group 2 or higher disease ▪ Adverse tumour characteristics: High-risk disease; intermediate risk disease with intraductal cribriform morphology	NR	**Germline testing** Advanced Part I mHSPCa, regardless of age and FH **Germline and somatic testing** Advanced Part II mCRPCa to identify DNA repair deficiency mutations and micro-instability status to inform prognosis, targeted therapies and counselling regarding family risk	NR
5. ESMO (2020) UK Clinical Practice Guidelines for diagnosis, treatment and follow-up	NA		**Germline testing** ▪ FH of cancer ▪ Pathogenic mutations in cancer-risk genes on tumour testing	**Strong/ Moderate** **Strong**	**Germline testing** ▪ FH of cancer ▪ Pathogenic mutations in cancer-risk genes on tumour testing ▪ mPCa **Somatic testing** ▪ mCRPCa	**Strong/ Moderate** **Strong** **Strong/ Moderate** **Strong/Moderate**
6. eviQ (Cancer Institute NSW) (2020) Australia Prostate cancer – panel testing (under review) 3648 v.3	**Germline testing** ▪ Unaffected known or obligate male pathogenic variant carrier of PCa associated genes ▪ Male at 50% risk of being pathogenic variant carrier	NR	**Germline testing** ▪ PCa and evidence of DNA MMR deficiency in tumour tissue ▪ PCa where a pathogenic variant in a listed gene has been detected on tumour testing ▪ PCa and Ashkenazi Jewish heritage.	NR	**Germline testing** ▪ PCa and evidence of DNA MMR deficiency in tumour tissue ▪ PCa where a pathogenic variant in a listed gene has been detected on tumour testing ▪ PCa and Ashkenazi Jewish heritage. **Somatic testing** ▪ high-risk localised or mPCa and ≥10% probability of detecting *BRCA1/BRCA2* pathogenic variant using validated prediction tool (e.g., CanRisk). ▪ mCRPCa with measurable metastatic disease diagnosed <60 y (regardless of other personal or family history)	NR
7. Advanced Prostate Cancer Consensus Conference (APCCC) Switzerland Management of Patients with Advanced Prostate Cancer (2018 & 2020)	NA		NA		**Germline testing** ▪ PCa diagnosed <60 y ▪ Positive FH of other cancer syndromes (HBOC and/or pancreatic Ca and/or LS) ▪ Newly diagnosed mPCa hormone sensitive/castration naive **Somatic testing** ▪ mPCa ▪ mCRPCa	NR

*PCa* prostate cancer, *NCCN* National Comprehensive Cancer Network, *close relative first* second and sometimes third degree relative, *FDR* first degree relative, *SDR* second degree relative, *TDR* third degree relative, *LS* Lynch Syndrome, *N1* advanced to nearby lymph nodes, *MSI-H* micro satellite instability-high, *MMR* mismatch repair genes, *TMB* tumour mutational burden, *HRR* homologous recombination repair genes, *mCRPCa* metastatic castrate resistant prostate cancer, *mPCa* metastatic prostate cancer, *EAU* European Association of Urology, *EANM* European Association of Nuclear Medicine, *ESTRO* European SocieTy of Radiology and Oncology, *ESUR* European Society of Urogenital Radiology, *ISUP* International Society of Urological Pathology, *SIOG* International Society of Geriatric Oncology, *csPCa* clinically significant prostate cancer, *mHSPC* metastatic hormone sensitive prostate cancer, *AUA* American Urological Association, *ASTRO* American Society of Radiation Oncology, *SUO* Society of Urologic Oncologists, *ESMO* European Society of Medical Oncologists, *NA* not applicable, *NR* not rated/expert opinion only.

^a^No prostate cancer.

^b^Cancer only inside prostate (I & II).

^c^Cancer outside prostate (III), and has spread to lymph nodes and other parts of the body (IV).

^d^Strength of recommendation ratings were mapped to the four National Health and Medical Research Council (NHMRC) grades of recommendation (A Strong: Body of evidence can be trusted to guide practice; B Strong/Moderate: Body of evidence can be trusted to guide practice in most situations; C Moderate: Some support for recommendation/s but care should be taken in its application; D Weak: Recommendation must be applied with caution).

## Results

The search generated 657 citations between the dates of January 1, 2007 to May 30, 2022. 102 duplicates were removed. The remaining 555 were imported into Covidence for title and abstract screening. 482 studies were excluded, leaving 73 studies for full text screening. Database alerts, collected between May 31, 2022 and August 5, 2022, generated three further guidelines for inclusion, bringing the total for full text screening to 76. After applying the PCC inclusion criteria to the full text screening, 23 guidelines and consensus statements from 16 different groups or organisations remained (Fig. 1).

### Research questions

A narrative summary, addressing each of the research questions in turn, accompanies the genetic testing strategies from each of the 23 included guidelines and consensus statements. Guidelines and consensus statements included in Table 3 were genetic testing guidelines or consensus statements from major organisations, recognised as authorities on the subject (*n* = 13). Major guidelines are thus defined as guidelines or consensus statements based on a clearly articulated process involving research evidence to support recommendations with consensus from a panel of experts from recognised medical organisations (national, or regional). The 10 remaining guidelines or consensus statements, are referred to as adapted guidelines, based on reviews of the major guidelines and consensus statements with country-specific, or other considered modifications based on specific stages of cancer, implementation, or practical clinical application. All adapted guidelines are also based on a rigorous methodology and consensus from a panel of experts. A summary table of these adapted guidelines is in Appendix III.

#### a. What genetic testing guidelines and consensus statements for prostate cancer currently exist?

Of the 13 major guidelines included in this review, six guidelines and two consensus statements were from organisations in the US, comprising the NCCN (*n* = 3) [21, 30, 31], a conglomerate of specialist prostate cancer clinician organisations (AUA; American Society for Radiotherapy and Oncology (ASTRO); Society of Urologic Oncology (SUO)) (*n* = 3) [32–34] and the Philadelphia Prostate Cancer Consensus Conference (*n* = 2) [16, 35]. Two guidelines and two consensus statements were from European organisations: ESMO; [23] a conglomerate of organisations comprising specialist prostate cancer clinicians (European Association of Urology (EAU), European Association of Nuclear Medicine (EANM), European Society for Radiotherapy and Oncology (ESTRO), European Society of Urogenital Radiology (ESUR), International Society of Geriatric Oncology (SIOG)) [22]; and the Advanced Prostate Cancer Society (APCCC) (*n* = 2) [36, 37]. One major guideline, eviQ, was from the Cancer Institute of NSW, Australia [38].

The ten remaining adapted guidelines comprised seven guidelines, two consensus statements and one position paper from various organisations in nine countries including Italy (Italian Scientific Societies) [39], France (Cancer Committee of the French Association of Urology (CCFAU)) [40], Spain (Spanish Society of Medical Oncology (SEOM) and Spanish Oncology Genitourinary Group (SOGUG)) [41], Canada (*n* = 2) (i. Canadian Consensus Forum [42] and ii. Canadian Expert Multidisciplinary Working Group in Genetic Testing for Metastatic Prostate Cancer [43]), Switzerland (Swiss Group for Clinical Cancer Research (SAKK) Network for Cancer Predisposition Testing and Counselling (CPTC)) [44], US (Large Urology Group Practice Association (LUGPA)) [45], Sweden (*n* = 2)(Swedish National Prostate Cancer Guidelines Group) [46, 47] and China (Hong Kong Urological Association and Hong Kong Society of Uro-Oncology) [48].

#### b. What are the recommendations for genetic testing of prostate cancer*?*

Genetic testing strategies from each of the major guidelines are summarised in Table 3. Genetic testing strategies from adapted guidelines are summarised in Appendix III.

#### c. Who should be considered for genetic testing?

All guidelines and consensus statements recommend genetic testing (germline and/or somatic) for men with metastatic prostate cancer. The NCCN guidelines offer the most detailed guidance across the three prostate cancer relevant guidelines included (Prostate Cancer; Genetic/Familial High-Risk Assessment: Breast, Ovarian and Pancreatic Cancer; and Colon Cancer). Essentially, germline testing is recommended for men with high- or very high-risk prostate cancer, regional or metastatic prostate cancer, regardless of family history. Germline testing is also recommended for men with a personal history of breast cancer or a positive family history of early onset breast, colorectal or endometrial cancer (age ≤50 years); ovarian, exocrine or pancreatic cancer (any age); prostate cancer ≤60 years or prostate cancer death; Lynch-syndrome related cancer, especially if diagnosed <50 years; or Ashkenazi Jewish ancestry.

Somatic testing is recommended for men with hormone sensitive metastatic prostate cancer or castrate resistant metastatic prostate cancer. While many of the major guidelines offer less specific and/or less comprehensive criteria for genetic testing than NCCN, all recommend germline and somatic testing for men with metastatic prostate cancer, particularly for men with personal or family history or Ashkenazi Jewish ancestry and early onset disease.

For men with early stage or localised prostate cancer, germline genetic testing is recommended only where it is likely to impact treatment, clinical trial options, risk management of other cancers and/or potential risk for family members. Testing criteria tend to focus on personal history of metastatic or high-risk prostate cancer, particularly early onset, and family history of prostate cancer, breast, ovarian, pancreatic, colorectal or endometrial cancer and Ashkenazi Jewish ancestry. Some guidelines [23, 38] recommend germline testing for men who have confirmed DNA MMR deficiency or a pathogenic variant in a listed gene after tumour testing. For this population, one guideline makes no recommendations [34], while others suggest genetic testing be considered only for men with personal or family history of high-risk germline mutations and/or early onset prostate cancer [44, 46, 48].

For men without prostate cancer, many guidelines make no mention of genetic testing [23, 34, 40] or make recommendations to consider germline testing for reasons of family history or ancestry [22, 38, 48], rather than recommending it. Germline testing is recommended for men without prostate cancer in the guidelines of only three organisations. NCCN recommend germline testing for men with a family history suggestive of hereditary prostate cancer or hereditary breast and ovarian cancer or colon cancer syndromes [21, 30, 31]. The Italian Scientific Societies recommend germline *BRCA* testing for men with a family history of hereditary breast or ovarian cancer or paternal family with breast or ovarian cancer [39]. The Spanish Society of Medical Oncology recommends germline testing for men with a family history of cancer predisposition [41].

#### d. Which genes should be tested for?

Men with prostate cancer may have germline mutations in a number of genes. Those genes with moderate to high risk hereditary cancer susceptibility include homologous recombination repair genes *BRCA2, BRCA1, CHEK2, ATM, PALB2, RAD51D;* mismatch repair genes *MLH1, MSH2, MSH6, PMS2;* and pathogenic variant *HOXB13*. These genes are implicated in a range of cancer types, with the exception of *HOXB13* which, to date, seems to be prostate cancer specific [12]. The NCCN provides the most comprehensive recommendations, recommending different genes for genetic testing based on the purpose of testing (Table 4) [21].

**Table 4:** Purpose of genetic testing and choice of genes.

Source Test	NCCN	ESMO	Philadelphia PCCC	AUA/ASTRO	EAU-EANM-ESTRO…	eviQ	APCCC
**Germline**	Increased risk PCa: HRR genes; *EPCAM*, MMR genes; Increased risk & early onset familial PCa: *HOXB13*; Early onset, aggressive phenotype, reduced survival: *BRCA2;* High risk HBOC syndrome: *BRCA1, BRCA2* pathogenic or likely pathogenic, *ATM*.	*BRCA1, BRCA2*, other DDR genes (e.g., *ATM, MSH2, FANCA, MLH1, RAD51B, RAD51C, CDK12, FANCD2*)	**No PCa**: High-risk for early detection: *BRCA2(r), HOXB13(r), BRCA1(c), ATM(c)*, MMR genes (particularly MSH2)(c);^a^ **Non-mPCa**: Active surveillance: BRCA2(r), ATM(c);^a^ **mPCa**: post somatic confirmatory testing for PCa disposition & cascade testing: *BRCA1, BRCA2, ATM*, MMR genes;^a^ precision therapy or clinical trial: broad germline testing *BRCA2, BRCA1, ATM* MMR genes^a^.	**Non-mPCa**: *ATM, BRCA1, BRCA2, CHEK2, HOXB13*, *MLH1, MSH2, MSH6, NBN, PALB2, PMS2, TP53;*^b^ **mPCa**: *BRCA2, ATM, CHEK2, BRCA1, RAD51D, PALB2;*^b^ **mCRPCa**: *ATM, BRCA1, BRCA2, PALB2;*^b^ inform prognosis, precision therapy and counselling re family risk MMR genes	*BRCA1, BRCA2, ATM, CHEK2, HOXB13*, MMR *genes;* High-risk: *BRCA2, ATM*, MMR genes (particularly MSH2).	*ATM, BRCA1, BRCA2, HOXB13*, *MMR* genes.^b^	Large panel testing to include HRR genes, MMR genes; After positive somatic testing for *BRCA1, BRCA2, HOXB13, ATM*, MMR genes.^c^
**Somatic**	precision therapy or clinical trial for: **mPCa:** *BRCA1, BRCA2, ATM, PALB2, FANCA, RAD51D, CHEK2, CDK12;* **mCRPCa**: MMR genes	HRR genes, MMR genes	**mPCa**: NGS testing; **mCRPCa**: Precision therapy- PARP inhibitors *BRCA2(r)*, *BRCA1(c);* Platinum-based chemotherapy *BRCA1(c), BRCA2(c);* Anti-PD1 MMR genes(c)	**mCRPCa**: inform prognosis, precision therapy and counselling re family risk MMR genes	**mPCa**: HRR genes; MMR genes (followed by germline for BRCA1, BRCA2, ATM, MMR genes); **mCRPCa**: somatic and/or germline as above	*ATM, BRCA1, BRCA2, HOXB13*, *MMR* genes.^b^	*BRCA1, BRCA2*, MMR genes.

*PCa* prostate cancer, *HRR* (homologous recombination repair) genes *BRCA1, BRCA2, ATM, PALB2, CHEK2;* MMR (mismatch repair) genes *MLH1, MSH2, MSH6, PMS2*, *mPCa* metastatic prostate cancer, *mCRPCa* metastatic castrate resistant prostate cancer, *NGS* next generation sequencing (comprehensive genetic testing), (*r*) recommend, (*c*) consider.

^a^Test additional genes on basis of personal or family history(r).

^b^none specified, PCa associated genes identified only.

^c^no consensus regarding type of germline testing.

Other guidelines base their recommendations on disease stage [22, 32–35] or a combination of both purpose and stage. While there is some consensus regarding which genes to test, recommendations across guidelines vary. For example, for metastatic castrate resistant prostate cancer, recommendations range from the type of test (germline and/or somatic) with no specific genes nominated [42, 47] or testing for one gene only (*BRCA2*) [47] compared to the more comprehensive list recommended by NCCN in Table 4 above. For those with high-risk or metastatic prostate cancer, one guideline recommends germline testing only after somatic testing or after a validated prediction tool (e.g., CanRisk) confirms a ≥ 10% probability of detecting BRCA1/2 pathogenic variant [38], whereas many guidelines and consensus statements recommend germline testing across a range of genes for all men diagnosed with metastatic prostate cancer [16, 21–23, 30–37, 41, 43, 45, 48].

#### e. Which testing methods are used and where are samples drawn from?

Few guidelines or consensus statements provide further specificity than germline and/or somatic testing in relation to testing methods or where samples are drawn from. Recommendations tend to range from targeted gene tests for one or two genes (*BRCA1/2*) to a prespecified gene panel (e.g. HRR and/or MMR genes) [16, 21, 30, 31, 35, 45], or large panel testing for advanced prostate cancer [36, 37]. Whole exome or whole genome sequencing was not mentioned in any of the included guidelines or consensus statements. Typically, germline testing samples blood or saliva and somatic testing samples the tumour or metastatic tissue. No guideline or consensus statement mentioned sampling plasma or testing for circulating tumour DNA. Putative mutations or variants of unknown significance (VUS) were mentioned only in relation to post-test counselling [16, 21, 30, 31, 35–37].

#### f. What evidence supports the recommendations?

All guidelines and consensus statements involved a review of the literature as an evidence base. While guidelines and consensus statements often employed different methods to rate the level of evidence or strength of recommendation to support their recommendations, in general, evidence was reported as lower level. For example, all included NCCN recommendations were rated 2a, meaning the guideline statement is based upon lower-level evidence, however, NCCN consensus is that the intervention is appropriate. Expert opinion, which comprised reviews of the literature and consensus panels, was cited as strength of recommendation in 10 guidelines [16, 32–39, 42–47]. Other guidelines and consensus statements used modified GRADE evidence ratings [22, 40, 48] had their own strength of evidence ratings [32–34] or grades of recommendation [23] or adopted other systems from previous clinical guidelines [41] to rate the strength of their recommendations.

## Discussion

This scoping review is the first systematic and comprehensive review to examine current worldwide guidelines and consensus statements for genetic testing of prostate cancer. While numerous guidelines and consensus statements exist and genetic testing is now routinely recommended for patients with prostate cancer, there is still considerable lack of consensus with regard to timing and the strategies for testing, even across more high income countries [49, 50]. As a consequence, there are differences across guidelines and consensus statements based on medical knowledge, available resources, as well as country of origin (including differences in health systems, workforce expertise and capacity, infrastructure, and so on). The synthesised evidence from this scoping review of 23 current guidelines and consensus statements will form the survey inputs from which a Delphi Panel will determine an evidence-based, stakeholder endorsed set of genetic testing strategies for prostate cancer. These strategies could be valuable for the development of local genetic testing guidelines or for the development of an international guideline. A standardised approach to genetic testing for prostate cancer is essential to establish the value of genetic testing for prostate cancer.

A number of points of contention with genetic testing guidelines and consensus statements have been raised in the literature and are discussed below. These concerns span the process from initiation of genetic testing or systematic identification of appropriate patients, pre-test counselling, education of clinicians and patients, informed consent, collection of family history, testing platforms, test selection and ordering, delivery of results and follow up, post-test counselling, and cascade testing, and include the need for practical strategies and flexibility in delivery as a response to health system challenges. Very few guidelines or consensus statements provide any guidance on, or consideration of, the impact of implementation of genetic testing [31, 35], nor do they consider such testing within the context of survivorship care [31]. For example, recommendations such as the strategy to offer germline genetic testing to all men diagnosed with metastatic prostate cancer would create implementation challenges and significant barriers for both providers and patients in the delivery of genetic testing, due simply to the number of men diagnosed, even in those countries where such recommendations are currently approved. With developments in genomics and targeted treatments, germline genetic testing is now routinely recommended in some countries for all men diagnosed with prostate cancer [50]. Integrating genetic testing into urology or oncology clinical workflows will thus require considerable planning and coordination if precision oncology is to realise the full benefits of genetic testing.

It is not just the challenges with genetic testing itself (availability of facilities to conduct testing, sufficient qualified staff to analyse tests and meet demand) that contributes to such challenges. Genetic counselling, while broadly accepted as a necessary part of the process of genetic testing can also be problematic. For example, some guidelines and consensus statements recommend genetic counselling pre and post genetic testing, along with a list of topics to be covered; others mention that genetic counselling is an essential and mandatory part of the genetic testing process but provide little other detail, and some make no mention of genetic counselling at all. The reality is that access to genetic counsellors is often very limited. Saad recently commented that, in Canada, where the government has approved genetic testing for metastatic prostate cancer at the time of diagnosis, it can take 6–12 months to see a genetic counsellor [49]. In Australia, a mainstream model of genetic testing for men with metastatic prostate cancer, where the oncologist is responsible for the counselling, consenting and ordering of the genetic testing, was found to be feasible, efficient and acceptable to both patienrts and clinicians [51].

While some guidelines or consensus statements [31, 35] provide a list of topics to be covered in genetic counselling, few raised the psychosocial issues associated with genetic testing, particularly for men with metastatic prostate cancer. One notable exception was the Swedish guidelines which cite concern for psychological impact on the patient and their family as well as insufficient evidence as reasons for their particularly conservative approach to genetic testing recommendations [47]. Moreover, given the increasing drive towards applying a survivorship care framework as a means of addressing fragmentation and gaps in prostate cancer care, situating genetic testing within such a framework presents as a priority [52]. This is an area that should be addressed in future research.

Another concern associated with genetic testing raised in the literature is one of equity. With access to genetic testing providers limited, it is unsurprising that most services, genetic counselling and genetic testing, are located in urban areas or academic institutions [43, 53]. This may exclude or make access difficult for patients in regional or rural areas. In lower and middle income countries, services may not exist or where countries do not provide health insurance or genetic testing free of charge, the expense of genetic testing may be prohibitive for many patients.

Prostate cancer is a common and heterogeneous disease and hereditary prostate cancer is an important clinical consideration with numerous epidemiological and hereditary risk factors. Further developments in genetic testing have the potential to advance the science around prostate cancer predisposition, just as personalised screening and testing can contribute to more accurate knowledge of the mechanisms of hereditary prostate cancer. While recent reviews of economic evaluations of breast, ovarian and colorectal cancer suggest genetic testing is likely to be cost effective for patients in some settings, currently, there is a lack of economic evaluation and cost-effectiveness evidence for genetic testing of prostate cancer [54, 55]. This evidence is imperative to inform who should be tested, how they should be tested and the most appropriate management pathway. Consensus or a standardised approach to genetic testing for prostate cancer is crucial to determining the value of genetic testing for prostate cancer. However, there is also recognition of a need for flexibility and innovation in delivery of genetic testing in countries and/or regions that do not have the resources to deliver genetic testing as per internationally or nationally recognised guidelines.

## Supplementary information

Appendix I: Protocol

Appendix II: Search strategy

Appendix III: Summary table of adapted genetic testing guidelines

## Data Availability

All data generated or analysed during this study are included in this published article and its supplementary information files.
