# Supplementary material for: Guidelines for genetic testing in prostate cancer: a scoping review
Source: Prostate Cancer Prostatic Dis. 2023 May 18;27(4):594–603. doi: 10.1038/s41391-023-00676-0 (PMC11543603; doi:10.1038/s41391-023-00676-0)
Supplement: Supplementary file 1 — Appendix I: Protocol [file 41391_2023_676_MOESM1_ESM.docx]

## Appendix I: Genetic testing in prostate cancer: a scoping review protocol

# Authors

Haitham Tuffaha*,^1^ Kim Edmunds,^1^ David Fairbairn,^2^Matthew J. Roberts,^3^ Suzanne Chambers,^4^ David P Smith,^5^ Lisa Horvath,^6^ Shiksha Arora,^1^ Paul Scuffham.^7^

1. Centre for the Business and Economics of Health, University of Queensland, Brisbane, Australia
2. Pathology Queensland, The Royal Brisbane Women’s Hospital, Brisbane, Australia
3. UQ Centre for Clinical Research, University of Queensland, Brisbane, Australia; Department of Urology, Royal Brisbane and Women’s Hospital, Brisbane, Australia
4. Australian Catholic University, Brisbane, Australia
5. The Daffodil Centre, The University of Sydney, A Joint Venture with Cancer Council NSW, Sydney, Australia

6. Medical Oncology, Chris O’Brien Lifehouse, Camperdown, New South Wales, Australia 7. Menzies Health Institute Queensland, Griffith University, Gold Coast, Australia

# Abstract

**Introduction:** Genetic testing, to identify pathogenic or likely pathogenic variants in prostate cancer, is valuable in guiding treatment decisions for the men with prostate cancer and to inform cancer prevention and early detection options for their blood family members who wish to learn about their own risks. There are, however, multiple recommendations and guidelines using different levels of evidence and uncertainty about how best to offer genetic testing in terms of who should be tested, which tests to use, and which set of strategies is most effective and best represents value for money.

**Objective:** To identify genetic testing recommendations in prostate cancer and the evidence supporting these recommendations for development of Australian recommendations.

**Methods:** The review will follow PRISMA-ScR guidelines and include literature searches in electronic databases and manual searches of grey literature and websites of key organisations. Guidelines and consensus statements published in English over the past ten years will be included. Strategies from these guidelines will be synthesised. Results will thus comprise a list of best available strategies for genetic testing and the evidence to support them.

**Inclusion criteria:** Using the Population, Concept, Context (PCC) framework, this scoping review will incorporate the following inclusions: prostate cancer patients or men at high risk of prostate cancer and their biological families; existing guidelines and supporting evidence for genetic testing of men with prostate cancer from any geographical location worldwide. Relevant exclusions are editorials, commentaries, abstracts and opinion pieces.

# Introduction

Prostate cancer (PCa) is the most common cancer type in Australian men (~21,000 cases diagnosed annually) and a leading cause of cancer-related deaths (~3,300 a year) (1, 2). The estimated annual cost of PCa treatment to Australia is approximately $500 million, and projected to increase by 40% over the next 10 years (3). Prostate cancer has a significant heritable component, with >50% of its risk attributed to genetic factors (4-6). Genetic testing, to identify pathogenic or likely pathogenic variants in PCa, is valuable to guide treatment decisions for men with PCa and to inform cancer prevention and early detection options for their blood family members who wish to learn about their own risks. The potential benefits of genetic testing, however, are not realised because there is substantial uncertainty about how best to offer genetic testing in terms of who should be tested and which tests to use.

Importantly, genetic testing in PCa predicts cancer risk and outcomes (7, 8). Several inherited pathogenic or likely pathogenic variants (e.g., *BRCA1, BRCA2, ATM, PALB2, CHEK2*) are associated with varying degrees of increased risk to PCa (4-6). Moreover, *BRCA1 and BRCA2* mutations are associated with a younger age of cancer onset and an aggressive clinical course with higher cancer mortality (8).

In addition, genetic testing could inform treatment decisions. Men diagnosed with PCa who carry a pathogenic or likely pathogenic variant could benefit from more frequent assessment and early treatment if they have early-stage disease. Men with metastatic (i.e., advanced) PCa could benefit from targeted therapies (e.g., PARP inhibitors) to prolong life and optimise treatment outcomes, including reduced adverse events (9, 10).

There is also potential for significant benefits from testing family members of carriers with a pathogenic or likely pathogenic variant (i.e., cascade testing) to guide early detection and prevention measures (11). For example, female relatives who test positive for a pathogenic or likely pathogenic *BRCA* variant are at higher risk of breast and/or ovarian cancer and may choose to undertake preventive strategies (e.g., surgery to remove breasts/ovaries) and consider family planning options (11). Male relatives who have a pathogenic or likely pathogenic variant could also be screened and assessed for heritable cancer (e.g., prostate and pancreatic) at a younger age than others to aid early detection and cure (11).

Clinical practice guidelines (e.g., National Comprehensive Cancer Network (NCCN)) recommend that genetic testing be offered to patients with metastatic prostate cancer, and to those who qualify for testing based on family history (12, 13). These guidelines are primarily based on expert opinion, and it is unclear how best to offer genetic testing in terms of which patients to test and which tests to use (9, 14, 15). There are potentially 5000 Australians who would benefit from genetic testing each year, but the cost would be prohibitive without Medicare reimbursement (1, 2). Given these current limitations, the rationale for this scoping review is to identify existing strategies for genetic testing of PCa and the evidence supporting those strategies in order to provide a comprehensive list of possible strategies for consideration by the Delphi Panel members. The overarching objective of this scoping review is to understand the evidence in relation to how best to conduct genetic testing of PCa.

A preliminary search of MEDLINE, the Cochrane Database of Systematic Reviews and *JBI Evidence Synthesis* was conducted and no current or underway systematic reviews or scoping reviews on genetic testing for PCa were identified.

# Review questions

1. What recommendations and guidelines currently exist for genetic testing of PCa?
2. What are the suggested strategies for genetic testing of PCa?
   1. Who should be considered for genetic testing?
   2. When do they get tested?
   3. Which genes should be tested for?
   4. Which testing methods are used (e.g., whole exome sequence, genome sequence, gene panels, genome-wide association study, prespecified genes to sequence and identify mutations (some of which will be known to be putative and others will be “variants of unknown significance” (VUS))?
   5. Where will the samples be drawn from (e.g., tumour vs blood for somatic; blood vs buccal or other tissue for germline)?
   6. What evidence supports each (clinical and/or economic) strategy?
   7. What impacts to clinical management are expected in supporting each strategy? (e.g., testing in at risk men for earlier diagnosis and cure; testing in low-risk men on active surveillance; testing in treated men at high risk of recurrence; testing in men with advanced cancer for prognosis + treatment eligibility such as PARPi)

# Keywords

guidelines; familial; genetic testing; germline; somatic; inherited; prostate cancer

# Eligibility criteria

### Participants

Prostate cancer patients or men at high risk of prostate cancer and their families.

### Concept

### Recommendations, guidelines, consensus statements and supporting evidence for genetic testing of men with prostate cancer.

### Context

### Any context where genetic testing for prostate cancer is possible. There are no specific cultural/sub-cultural factors, geographical locations, specific racial or gender-based considerations. The context must be applicable to the Australian context.

### Types of Sources

This scoping review will consider all recommendations, guidelines and consensus statements and relevant supporting evidence reported in the guidelines. Reviews will be incorporated where they are conducted by a consortium or multidisciplinary national or international team in order to develop country/region specific guidelines or to advance clinical application or implementation of guidelines. Grey literature such as unpublished guidelines or those published informally or non-commercially will also be included in the scoping review, providing a clear methodology incorporating clinical experts and evidence-based decisions is presented.

Published papers such as opinion pieces, commentaries, editorials and conference abstracts will not be included in this scoping review.

# Methods

The review will follow PRISMA-ScR guidelines and include literature searches in electronic databases PubMed, Embase and CINAHL and manual searches of grey literature and the websites of key organisations (e.g., eviQ, NCCN, Philadelphia statement, ESMO) (16). Guidelines and consensus statements published in English over the past fifteen years will be included. Strategies from these will be synthesised into a comprehensive list. Results will thus comprise a list of strategies for genetic testing and the evidence to support them, from which a Delphi panel will determine an evidence-based, stakeholder endorsed set of genetic testing strategies for PCa.

### Search strategy

The search strategy will aim to locate both published and unpublished guidelines. An initial limited search of Medline and CINAHL was undertaken to identify articles on the topic of genetic testing for prostate cancer. The text words contained in the titles and abstracts of relevant articles, and the index terms used to describe the articles were used to develop a full search strategy for genetic testing guidelines for PCa (see Appendix 1). The search strategy, including all identified keywords and index terms, will be adapted for each included database and/or information source. The reference list of all included sources of evidence will be screened for additional studies. Guidelines and recommendation statements published since April 1, 2007, when the first genome wide association study for PCa was published, until May 30 2022, will be included to ensure all possible guidelines and associated evidence are captured (17).

The databases to be searched include PubMed, Embase and CINAHL. Sources of unpublished studies/grey literature to be searched include the Cancer Institute NSW eviQ, National Comprehensive Cancer Network (NCCN), the Philadelphia statement and the European Society for Medical Oncology (ESMO).

### Study/Source of Evidence selection

Following the search, all identified citations will be collated and uploaded into Endnote 20 *(Clarivate Analytics, PA, USA)* and duplicates removed. The Endnote file will then be uploaded into Covidence (<https://www.covidence.org/>). Following a pilot test, titles and abstracts will then be screened by two independent reviewers for assessment against the inclusion criteria for the review. Potentially relevant sources will be retrieved in full. The full text of selected citations will be assessed in detail against the inclusion criteria by two independent reviewers. Reasons for exclusion of sources of evidence at full text that do not meet the inclusion criteria will be recorded and reported in the scoping review. Any disagreements that arise between the reviewers at each stage of the selection process will be resolved through discussion, or with an additional reviewer. The results of the search and the study inclusion process will be reported in full in the final scoping review and presented in a Preferred Reporting Items for Systematic Reviews and Meta-analyses extension for scoping review (PRISMA-ScR) flow diagram (18).

### Data Extraction

Data will be extracted from papers included in the scoping review by independent reviewers using a data extraction tool developed by the reviewers. The data extracted will include specific details about the guideline: title, version, organisation, recommendations; implications, evidence and level of evidence.

A draft extraction form is provided (see Appendix II*).*The draft data extraction tool will be modified and revised as necessary during the process of extracting data from each included evidence source. Modifications will be detailed in the scoping review. Any disagreements that arise between the reviewers will be resolved through discussion, or with an additional reviewer.

### Data Analysis and Presentation

# Genetic testing strategies and the evidence that supports them will be presented in tabular form. A narrative summary will accompany the tabulated results and will describe how the results relate to the review’s objective and research questions.

# Funding

This scoping review was funded via a grant from the Prostate Cancer Foundation of Australia. The funders were not involved in the review process. Several members of the PCFA are authors on this paper who contributed their specific expertise regarding genetic testing of PCa.

# Conflicts of interest

There is no conflict of interest in this project*.*

# References

1. Australian Institute of Health and Welfare. Cancer data in Australia. 2021.

2. Department of Health. Prostate cancer screening-position statement. 2019.

3. Gordon LG TH, James R, Keller AT, Lowe A, Scuffham PA, et al. 36(3):91.e7–.e15. Estimating the healthcare costs of treating prostate cancer in Australia: A Markov modelling analysis. Urological Oncology. 2018;36(3):91.e7-.e15.

4. Crumbaker M WJ, Joshua AM, Spigelman AD. Asia Pac J Clin Oncol. 2019;. Outcomes of universal germline testing for men with prostate cancer in an Australian tertiary center. Asia Pac J Clin Oncol. 2019;15:257–61.

5. Nicolosi P, Ledet E, Yang S, Michalski S, Freschi B, O’Leary Eea. Prevalence of germline variants in prostate cancer and implications for current genetic testing guidelines. . JAMA Oncology. 2019;5(4):523-8.

6. Pritchard C, Mateo J, Walsh M, De Sarkar N, Abida W, Beltran Hea. Inherited DNA-repair gene mutations in men with metastatic prostate cancer. . N Engl J Med. 2016;375(5):443-53.

7. Castro E, Goh C, Olmos D, Saunders E, Leongamornlert D, Tymrakiewicz M, et al. Germline BRCA mutations are associated with higher risk of nodal involvement, distant metastasis, and poor survival outcomes in prostate cancer. J Clin Oncol. 2013;31(14):1748-57.

8. Giri V, Knudsen K, Kelly W, Abida W, Andriole G, Bangma C, et al. Role of Genetic Testing for Inherited Prostate Cancer Risk: Philadelphia Prostate Cancer Consensus Conference 2017. Journal of Clinical Oncology. 2018;36(4):414-24.

9. Cheng H, Powers J, Schaffer K, Sartor O. Practical methods for integrating genetic testing into clinical practice for advanced prostate cancer. . Am Soc Clin Oncol. 2018;38:372-81.

10. de Bono J, Mateo J, Fizazi K, Saad F, Shore N, Sandhu S, et al. Olaparib for Metastatic Castration-Resistant Prostate Cancer. N Engl J Med. 2020;382(22):2091-102.

11. Le D, Uram J, Wang H, Bartlett B, Kemberling H, Eyring A, et al. PD-1 blockade in tumors with mismatch-repair deficiency. . N Engl J Med. 2015;372(26):2509-20.

12. National Comprehensive Cancer Network. Genetic/Familial high-risk assessment: Breast, ovarian, and pancreatic: NCCN.org; 2022 [updated March 9. Version 2:[

13. National Comprehensive Cancer Network. Prostate Cancer: NCCN; 2022 [Version 3:[

14. Giri V, Knudsen K, Kelly W, Cheng H, Cooney K, Cookson MS ea, editors. Implementation of Germline Testing for Prostate Cancer: Philadelphia Prostate Cancer Consensus Conference 2019. Philadelphia Prostate Cancer Consensus Conference; 2019 2020.

15. Margo J. The hidden value of genetic testing for prostate cancer. Australian Financial Review 2019.

16. Rethlefsen M, Kirtley S, Waffenschmidt S, Ayala A, Moher D, Page M, et al. PRISMA-S: an extension to the PRISMA Statement for Reporting Literature Searches in Systematic Reviews. Systematic Reviews 2021;10(39).

17. Hsu C-C, Sandford B. The Delphi Technique: Making sense of consensus. . Pract Assess Res Eval. 2007;12(10):1-8.

18. Tricco A, Lillie E, Zarin W, O'Brien K, Colquhoun H, Levac D, et al. PRISMA Extension for Scoping Reviews (PRISMA-ScR): Checklist and Explanation. Annals of Internal Medicine. 2018;169:467-73

# Appendices

### Appendix I: Search strategy

This search strategy will be replicated with necessary database specific modifications for Embase and CINAHL databases

## Updated Search date: 13/06/2022

("Prostatic Neoplasms"[Mesh] OR (prostat*[tiab] AND (cancer*[tiab] or neoplas*[tiab] or tum?r[tiab] or carcinoma*[tiab])))

AND

("Genetic Testing"[Mesh] OR "Genetic Counseling"[Mesh] OR "Genetic Carrier Screening"[Mesh] OR "Genetic Services"[Mesh] OR ((germline*[tiab] OR somatic*[tiab] OR genetic*[tiab]) AND (screen*[tiab] OR test*[tiab] OR panel*[tiab])))

AND

((clinical[tiab] AND pathway*[tiab]) OR (care[tiab] AND pathway*[tiab]) OR guideline[tiab] OR guidance[tiab] OR "Consensus"[Mesh] OR "Consensus Development Conference, NIH" [Publication Type] OR "Consensus Development Conference" [Publication Type] OR "Consensus Development Conferences, NIH as Topic"[Mesh] OR "Consensus Development Conferences as Topic"[Mesh] OR "Critical Pathways"[Mesh] OR "Guidelines as Topic"[Mesh] OR "Practice Guidelines as Topic"[Mesh] OR "Health Planning Guidelines"[Mesh] OR "Practice Guideline" [Publication Type])

AND

(2007/1/1:2022/5/30[pdat])

## Results:

PubMed – 190

EMBASE – 371

CINHAL – 62

PsycInfo – No articles found

### Appendix II: Draft Data extraction instrument

| **Genetic Testing for PCa** | | | | **Pretesting****Target population** | | | | | **Genetic Testing** | | | **Management implications** | | | **Evidence** | | |
| --- | --- | --- | --- | --- | --- | --- | --- | --- | --- | --- | --- | --- | --- | --- | --- | --- | --- |
|  |  |  |  | **Criteria for testing** | | | | |  |  |  |  |  |  |  |  |  |
| **#** | **First author****(Year)****Country** | **Guideline title** | **Guideline type (publication; website…)** | **Men without PCa** | **Men with low risk/localised PCa** | **Men with high risk localised/ advanced PCa** | **Family History** | **Counselling** | **Which testing methods are used?** | **Which genes should be tested for (familial/ clinical testing)?** | **Where will the samples be drawn from?** | **Clinical consequences (how do results inform management? e.g.; screening/treatment)** | **Expected public health benefits** | **Comment/critique in guideline** | **Source** | **Grading of evidence** | **Other comments** |
