# Supplementary material for: Guidelines for genetic testing in prostate cancer: a scoping review
Source: Prostate Cancer Prostatic Dis. 2023 May 18;27(4):594–603. doi: 10.1038/s41391-023-00676-0 (PMC11543603; doi:10.1038/s41391-023-00676-0)
Supplement: Supplementary file 2 — Appendix II: Search strategy [file 41391_2023_676_MOESM2_ESM.docx]

### **Appendix II: Search strategy**

This search strategy will be replicated with necessary database specific modifications for Embase and CINAHL databases

## Updated Search date: 13/06/2022

("Prostatic Neoplasms"[Mesh] OR (prostat*[tiab] AND (cancer*[tiab] or neoplas*[tiab] or tum?r[tiab] or carcinoma*[tiab])))

AND

("Genetic Testing"[Mesh] OR "Genetic Counseling"[Mesh] OR "Genetic Carrier Screening"[Mesh] OR "Genetic Services"[Mesh] OR ((germline*[tiab] OR somatic*[tiab] OR genetic*[tiab]) AND (screen*[tiab] OR test*[tiab] OR panel*[tiab])))

AND

((clinical[tiab] AND pathway*[tiab]) OR (care[tiab] AND pathway*[tiab]) OR guideline[tiab] OR guidance[tiab] OR "Consensus"[Mesh] OR "Consensus Development Conference, NIH" [Publication Type] OR "Consensus Development Conference" [Publication Type] OR "Consensus Development Conferences, NIH as Topic"[Mesh] OR "Consensus Development Conferences as Topic"[Mesh] OR "Critical Pathways"[Mesh] OR "Guidelines as Topic"[Mesh] OR "Practice Guidelines as Topic"[Mesh] OR "Health Planning Guidelines"[Mesh] OR "Practice Guideline" [Publication Type])

AND

(2007/1/1:2022/5/30[pdat])

## Results:

PubMed – 190

EMBASE – 371

CINHAL – 62

PsycInfo – No articles found

Papers included after original search up to August 5, 2022 based on database alerts
