# Supplementary material for: Guidelines for genetic testing in prostate cancer: a scoping review
Source: Prostate Cancer Prostatic Dis. 2023 May 18;27(4):594–603. doi: 10.1038/s41391-023-00676-0 (PMC11543603; doi:10.1038/s41391-023-00676-0)
Supplement: Supplementary file 3 — Appendix III: Summary table of adapted genetic testing guidelines [file 41391_2023_676_MOESM3_ESM.docx]

**Appendix III: Summary table adapted genetic testing guidelines**

| **Guideline organisation**  **Title**  **(Year) Country** | **Criteria for genetic testing by cancer diagnosis** | | | | | | **Test type and genes** |
| --- | --- | --- | --- | --- | --- | --- | --- |
|  | **Men without PCa**  **(Stage 0)^1^** | **Strength of Recom-mendation** | **Men with localised PCa (Stage I or II)^2^** | **Strength of Recom-mendation** | **Men with locally advanced or advanced PCa**  **(Stage III or IV)^3^** | **Strength of Recom-mendation** |  |
| **Adapted guidelines** | | | | | | | |
| 1. Italian Scientific Societies  Italy (2022)  Implementation of preventive and predictive *BRCA* testing in patients with breast, ovarian, pancreatic and prostate cancer: a position paper of Italian Scientific Societies | **Germline Testing**   - Personal history of male breast cancer or metastatic pancreatic cancer - FDR with HBOC genetic predisposition - SDR (paternal side) with breast or ovarian cancer | NR | **Germline Testing**  PCa and:   - at least one first-degree relative with prostate cancer aged <60 years - ≥2 family members with prostate cancer aged <50 years - FDR with HBOC genetic predisposition - SDR (paternal side) with breast or ovarian cancer | NR | **Germline Testing**   - **mPCa** - PCa and: - ≥1 FDR with prostate cancer aged <60 years - ≥2 family members with prostate cancer aged <50 years - FDR with HBOC genetic predisposition - SDR (paternal side) with breast or ovarian cancer   **Somatic testing**   - **mPCa** | NR | *BRCA* only in this guideline |
| 2. Cancer Committee of the French Association of Urology (CCFAU)  France (2022)  Cancer Committee of the French Association of Urology guidelines – update 2020-2022: prostate cancer | Oncogenetic Consultation   - Men with *BRCA2* or *HOXB13* gene mutation screened for PCa | **Strong** | Oncogenetic Consultation   - PCa <50 years; - Hereditary PCa: - PCa in 3 FDRs of the same family branch: (fathers, sons, brothers) or 2 SDRs (nephews, uncles-maternal or paternal) - PCa diagnosed at <55 years, in 2 FDRs (fathers, sons, brothers) or SDRs (nephews, uncles-maternal or paternal) - Family predisposition to HBOC: - 1 case breast cancer <40 y; - 1 case bilateral breast cancer; - 1 case ovarian cancer; - 1 case male breast cancer | **Strong**  **Strong**  **Strong** | Oncogenetic Consultation   - PCa <50 years; - Hereditary PCa: - PCa in 3 FDRs of the same family branch: (fathers, sons, brothers) or 2 SDRs (nephews, uncles-maternal or paternal) - PCa diagnosed at <55 years, in 2 FDRs (fathers, sons, brothers) or SDRs (nephews, uncles-maternal or paternal). - Family predisposition to HBOC: - 1 case breast cancer <40 y; - 1 case bilateral breast cancer; - 1 case ovarian cancer; - 1 case male breast cancer | **Strong**  **Strong**  **Strong** | No mention of testing type  Working group currently defining conditions for genetic testing re eligibility for treatment of **mPCa** (e.g., PARPi) |
| 3. Spanish Society of Medical Oncology (SEOM) and Spanish Oncology Genitourinary Group (SOGUG)  Spain (2021)  SEOM clinical guidelines for the treatment of advanced prostate cancer (2020) | **Germline testing**  Men with FH of cancer predisposition syndromes (e.g., breast, ovarian, pancreatic, LS) | **Strong** | **Germline Testing**  Men with FH of cancer predisposition syndromes for (e.g., breast, ovarian, pancreatic, LS) | **Strong** | **Somatic testing**   - **mPCa** - **mCRPCa**   **Germline testing**   - *BRCA1/BRCA2* and other genes associated with cancer predisposition syndromes - Men with somatic pathogenic/likely pathogenic mutations in genes linked to cancer predisposition syndromes (e.g., *BRCA2*). - Genetic counselling by clinicians with specific training or expertise before ordering germline testing. | **Strong**  **Strong**  **Strong**  **Strong**  **Strong** | **Germline and somatic**  *BRCA1 & BRCA2;* other genes as evidence emerges **Strong**  Broad panel including other HRR and MMR genes (and/or perform MSI assessment)  **Strong /Moderate** |
| 4. Canadian Consensus Forum Canada (2020)  Controversial issues in the management of patients with advanced prostate cancer: Results from a Canadian Consensus Forum | NA |  | NA |  | **Germline and/or somatic testing**   - Genetic counselling and testing in a minority of selected men with newly diagnosed mPCa. - Newly diagnosed mPCa with FH of PCa or other cancer syndromes (e.g., HBOC, pancreatic cancer, Lynch syndrome). - Newly diagnosed mPCa with positive FH of other cancers when:  ◦ men <60 years of age at diagnosis;   ◦ patients with visceral metastases;  ◦ those with intraductal or cribriform pathology. | NR | **Germline and/or somatic testing** (genes not specified) |
| 5. Swiss Group for Clinical Cancer Research (SAKK) Network for Cancer Predisposition Testing and Counselling (CPTC)  Switzerland (2021)  Update Swiss guideline for counselling and testing for predisposition to breast, ovarian, pancreatic and prostate cancer | Genetic counselling and **germline testing**   - Individual from family with known pathogenic variant in *BRCA1, BRCA2* or other gene conferring high/moderate risk for breast and/or ovarian cancer      - ≥1 close relative with breast or ovarian cancer and one of following: - ≤40 y - Bilateral ≤50 y - Age at diagnosis ≤50 y and:   - 1 close relative with breast cancer ≤50 y /unknown or limited FH   - Any age at diagnosis and:   - ≥2 close relatives with breast cancer  - close male relative with breast cancer  - ≥1 close relative with ovarian or pancreatic or metastatic/intraductal/cribriform PCa at any age | NR | Genetic counselling and **germline testing**   - Individual from family with known pathogenic variant in *BRCA1, BRCA2* or other gene conferring high/moderate risk for breast and/or ovarian cancer | NR | **Germline and somatic testing**   - Metastatic, intraductal or cribriform PCa at any age (first step: tumour profiling); - High grade (Gleason score ≥7) PCa and: - Ashkenazi Jewish ancestry; - 1 close relative with breast, ovarian, pancreatic or metastatic /intraductal /cribriform PCa; - ≥2 close relatives with breast or prostate cancer at any age | NR | **Germline and somatic testing** *BRCA1 & BRCA2* only; PCa relevant genes mentioned, but multi-gene panels for all HBOC cancers under development |
| 6. Large Urology Group Practice Association (LUGPA)  US (2020)  Optimizing the management of castration-resistant prostate cancer patients: A practical guide for clinicians | NA |  | NA |  | **Germline and/or Somatic testing**   - regional (any T, N1, M0) or metastatic (any T, any N, M1) PCa; - nodal or distant metastases - Genetic counselling as integral to genetic testing - Education in genetic testing for medical providers involved in management of advanced PCa. | NR | - MMR genes (Lynch syndrome); - HRR genes *BRCA2, BRCA1, ATM, PALB2, CHEK2;* - Cancer predisposition next‐generation sequencing (at a minimum) *BRCA2, BRCA1, ATM, CHEK2, PALB2,* MMR genes.   Additional genes depending on clinical context. |
| 7. Swedish National Prostate Cancer Guidelines Group  Sweden (2022)  The Swedish national guidelines on prostate cancer **Part 1:** early detection, diagnostics, staging, patient support, and primary management of non-metastatic disease  **Part 2:** recurrent, metastatic and castration resistant disease | NA |  | NA |  | **Germline testing**   - <60 years with mPCa or Gleason pattern 5 PCa and a first-degree relative with *BRCA2*-associated cancer      - Men with FH suggesting LS   **Somatic testing**   - **mCRPCa** | NR | **Germline testing**  *BRCA2*  **Somatic testing**  **mCRPCa**: *BRCA1* or *BRCA2* for PARPi therapy |
| 8. Canadian Expert Multidisciplinary Working Group in Genetic Testing for Metastatic Prostate Cancer  Canada (2022)  Recommendations for the implementation of genetic testing for metastatic prostate cancer patients in Canada | NA |  | NA |  | **Germline testing**   - Inform both familial cancer risk and treatment and clinical trial options   **Somatic testing**   - **mPCa** - **mCRPCa** | NR | **Germline testing**  **mPCa**: *ATM, BRCA1, BRCA2, CHEK2*, large deletions in *EPCAM, HOXB13, PALB2*, and MMR genes (aligns with NCCN, ESMO & Philadelphia). Additional genes depending on patient’s personal or FH.  **Somatic testing**  **mPCa**: *BRCA1, BRCA2, ATM, PALB2, FANCA, RAD51D, CHEK2, CDK12.*  **mCRPCa**: MSI-H or MMR genes, as clinically indicated, to include: *MLHI, MSH2, MSH6, PMS2, EPCAM.* |
| 9. Hong Kong Urological Association and Hong Kong Society of Uro-Oncology  China (2022)  Genetic Testing and its Clinical Application in Prostate Cancer Management: Consensus Statements from the Hong Kong Urological Association and Hong Kong Society of Uro-Oncology | NA |  | **Germline testing**   - Positive FH or patient is otherwise suspected of inherited PCa. - Ductal or intraductal histology - Tissue-based genetic assays for detailed risk assessment in localized PCa, and for counselling patients on AS or treatment | **Strong**  **Strong**  **Strong** | **Germline testing**   - Metastatic disease - Ductal or intraductal histology - FH positive or patient is otherwise suspected of inherited PCa - Confirmation of variants detected in somatic testing (particularly HRR or MMR genes)   **Somatic testing**   - **mHSPCa -** prognostic counselling and longer-term treatment planning - **mCRPCa -** actionable genetic mutations - Informed consent and genetic counselling coupled with genetic testing | **Strong**  **Strong**  **Strong**  **Strong**  **Strong**  **Strong**  **Strong** | **Germline testing**  HRR genes (*BRCA1/2, ATM, PALB2*) and MMR genes  **Somatic testing**  **mHSPCa:** genes involved in HRR (*BRCA1 & BRCA2*)  **mCRPCa:** Genetic testing can guide use of systemic therapies:  Anti-tumour activity with platinum-based chemotherapy or with PARPi in men with HRR mutations;  Anti-tumour activity with immune-checkpoint inhibitors for men with MSI-H or MMR tumors |
| **Abbreviations:** PCa prostate cancer; NCCN National Comprehensive Cancer Network; close relative first, second and sometimes third degree relative; FDR first degree relative; SDR second degree relative; LS Lynch Syndrome; N1 advanced to nearby lymph nodes; MSI-H micro satellite instability-high; MMR mismatch repair genes; HRR homologous recombination repair; mCRPCa metastatic castrate resistant prostate cancer; mPCa metastatic prostate cancer; M1 metastatic prostate cancer; mHSPCa metastatic hormone sensitive prostate cancer; AS active surveillance; ESMO European Society of Medical Oncologists; NA not applicable; NR not rated/expert opinion only; PARPi poly ADP-ribose polymerase inhibitors.  **Notes: ^1^** No prostate cancer; **^2^** Cancer only inside prostate (I & II)**; ^3^** Cancer outside prostate (III), and has spread to lymph nodes and other parts of the body (IV); ^4^ In order Strength of recommendation ratings were mapped to the four National Health and Medical Research Council (NHMRC) grades of recommendation (A Strong: Body of evidence can be trusted to guide practice; B Strong/Moderate: Body of evidence can be trusted to guide practice in most situations; C Moderate: Some support for recommendation/s but care should be taken in its application; D Weak: Recommendation must be applied with caution). | | | | | | | |
